# Axl Is Essential for *in-vitro* Angiogenesis Induced by Vitreous From Patients With Proliferative Diabetic Retinopathy

**DOI:** 10.3389/fmed.2021.787150

**Published:** 2021-12-23

**Authors:** Wenyi Wu, Huizuo Xu, Zhishang Meng, Jianxi Zhu, Siqi Xiong, Xiaobo Xia, Hetian Lei

**Affiliations:** ^1^Department of Ophthalmology, National Clinical Research Center for Geriatric Disorders, Xiangya Hospital of Central South University, Changsha, China; ^2^Department of Ophthalmology, Hunan Key Laboratory of Ophthalmology, Changsha, China; ^3^Department of Ophthalmology, The Second Xiangya Hospital of Central South University, Changsha, China; ^4^Department of Orthopedics, Xiangya Hospital of Central South University, Changsha, China; ^5^Department of Ophthalmology, Shenzhen Eye Institute, Shenzhen Eye Hospital, Jinan University, Shenzhen, China

**Keywords:** PDR vitreous, GAS6, Axl, CRISPR/Cas9, R428, HRECs

## Abstract

Proliferative diabetic retinopathy (PDR), characterized mainly with abnormal epiretinal angiogenesis forming fibrovascular membranes (FVMs), threatens vision of people with diabetes; FVMs consist of extracellular matrix and a variety of cell types including vascular endothelial cells. Axl, one of receptor tyrosine kinases, can be activated indirectly by vascular endothelial growth factor-A (VEGF-A) *via* an intracellular route for promoting angiogenesis. In this study, we revealed that growth arrest-specific protein 6 (Gas6), a specific ligand of Axl, was elevated in vitreous from patients with PDR and that Axl was activated in FVMs from patients with PDR. In addition, we demonstrated that in cultured human retinal microvascular endothelial cells (HRECs), Axl inhibition *via* suppression of Axl expression with Clustered Regularly Interspaced Short Palindromic Repeats/ CRISPR-associated protein 9 or through inactivation with its specific inhibitor R428 blocked PDR vitreous-induced Akt activation and proliferation of HRECs. Furthermore, PDR vitreous-heightened migration and tube formation of HRECs were also blunted by restraining Axl. These results indicate that in the pathogenesis of PDR, Axl can be activated by Gas6 binding directly and by VEGF-A *via* an intracellular route indirectly, suggesting that Axl plays a pivotal role in the development of PDR and that Axl inhibition shows a bright promise for PDR therapy.

## Introduction

Diabetic retinopathy (DR) is one of diabetic complications that affects eyes and proliferative DR (PDR) is the serious stage of DR. It is caused by damage to retinal microvascular endothelial cells, leading to vision-threatening exudation and hemorrhage. Retinal angiogenesis is a key pathological cause for PDR (1), and current PDR therapy mainly focuses on laser and antivascular endothelial growth factor (VEGF) drugs. However, photocoagulation has limited efficacy and acts as a devastating method; some patients poorly respond to current anti-VEGF therapy, but factors contributing to the limited response remain large. Further studies to identify and understand the molecular alterations that frequently occur in DR are vital to develop additional effective treatment options.

Vascular endothelial growth factor-A (VEGF-A) stimulates survival, proliferation, and migration of vascular endothelial cells (ECs) and promotes vascular permeability, which is critical for vascular development and angiogenesis. Due to its essential role in angiogenesis, VEGF-A signaling pathway has received great attention for angiogenesis therapeutics in the past decade. However, other growth factors also engage similar downstream signaling pathways including the phosphatidylinositol 3-kinase/ protein kinase B pathway to promote angiogenesis.

Axl, whose name came from the Greek word “anexelekto” meaning uncontrolled, is a member of the family of receptor tyrosine kinase (RTK). Its activation is stimulated by binding to its specific ligand growth arrest-specific protein 6 (GAS6) (2). In addition, Axl is also activated indirectly by VEGF-A *via* an intracellular pathway of reactive oxygen species (ROS)/Src family kinases (SFKs) (3). Furthermore, Axl has been found to confer resistance to anti-insulin-like growth factor 1 receptor (4); Fibroblast growth factor receptor 1-induced Akt activation is associated with Axl activity (5). Notably, Axl null mice respond poorly to VEGF-A-induced vascular permeability and angiogenesis (3). We previously showed that the normal bovine vitreous promotes endothelial cell proliferation and migration *via* Axl activation (6); however, whether Axl was activated in epiretinal fibrovascular membranes from patients with PDR remained elusive and the Axl role in human retinal vascular endothelial cells (HRECs) stimulated by PDR also needs to be clarified; for these answers, it will improve our understanding of the underlining mechanisms of pathogenesis of PDR and advance the development of additional effective treatment strategies for PDR.

## Results

### Axl Is Activated in Fibrovascular Membranes From Patients With PDR

**Figure 1:**
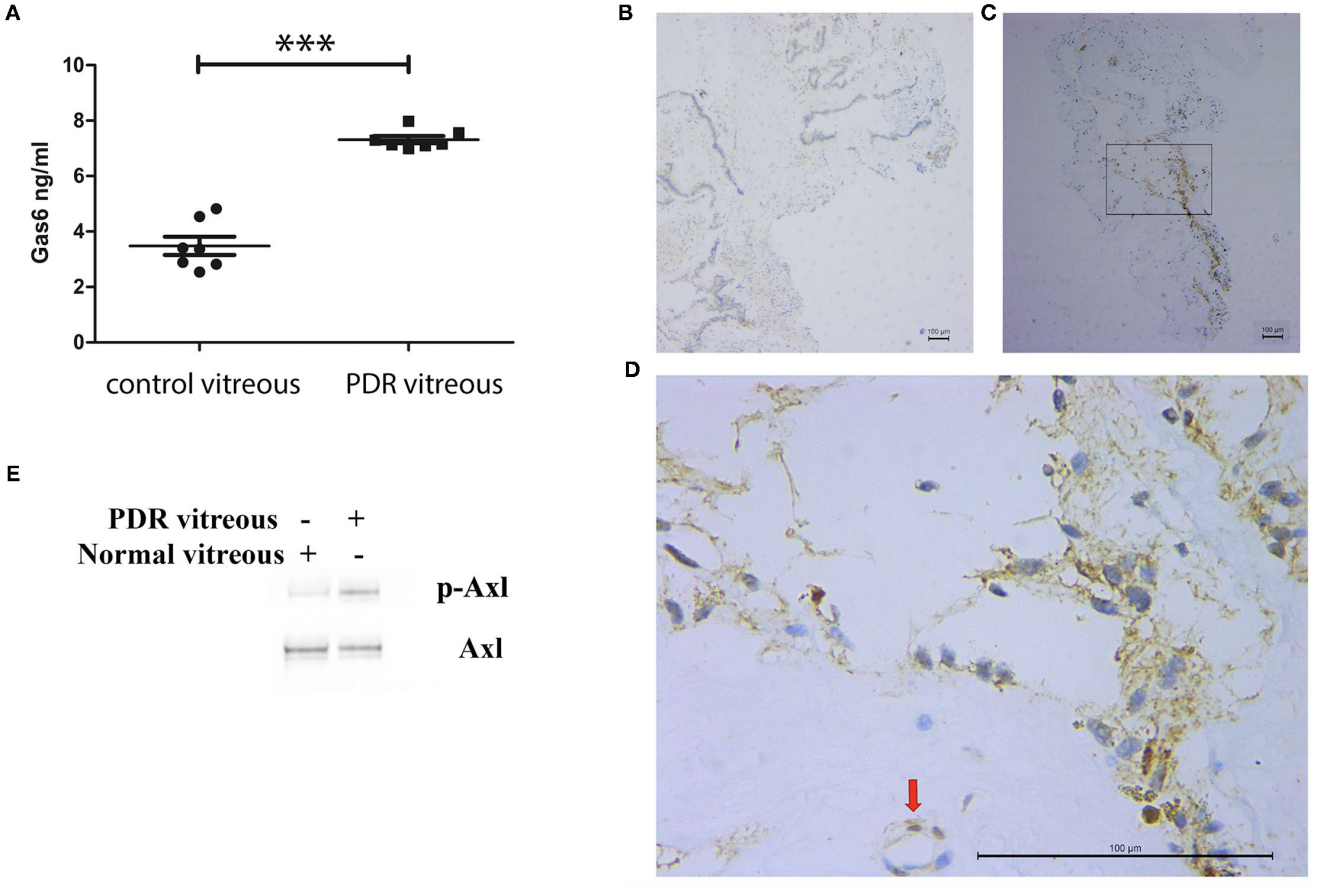
Elevation of growth arrest-specific protein 6 (Gas6), an Axl ligand in vitreous from patients with proliferative diabetic retinopathy (PDR). **(A)** ELISA analysis of Gas6 in vitreous from patients with or without PDR. **(B–D)** Immunohistochemistry analysis of p-Axl at fibrovascular membranes from patients with PDR. **(B)** Immunoglobulin G (IgG) as a negative control; **(C)** p-Axl; **(D)** higher magnification from **(C)**. **(E)** Western blot analysis of p-Axl in human retinal microvascular endothelial cells (HRECs) stimulated by PDR vitreous. ***means the difference was significant and *P* < 0.001.

Inhibition of Axl with a pharmacological inhibitor suppressed retinal angiogenesis in a mouse model of oxygen-induced retinopathy (6). To study if this finding was related to a clinical significance, we investigated if Gas6 was enriched in vitreous from patients with PDR by ELISA. In this assay, vitreous from the macular hole was used as a control. As shown in Figure 1A, in PDR vitreous, Gas6 was elevated compared with that in the control vitreous. Next, we evaluated if Axl was activated in fibrovascular membranes from patients with PDR by immunohistochemistry. The results (Figures 1B–E) showed that Axl was indeed activated in the PDR membranes, suggesting that Gas6 in the PDR vitreous is one of the possible agents inducing Axl activation in the PDR membranes.

### Proliferative Diabetic Retinopathy Vitreous Heightens Activation of Axl and Akt and Cellular Events Related to Angiogenesis

**Figure 2:**
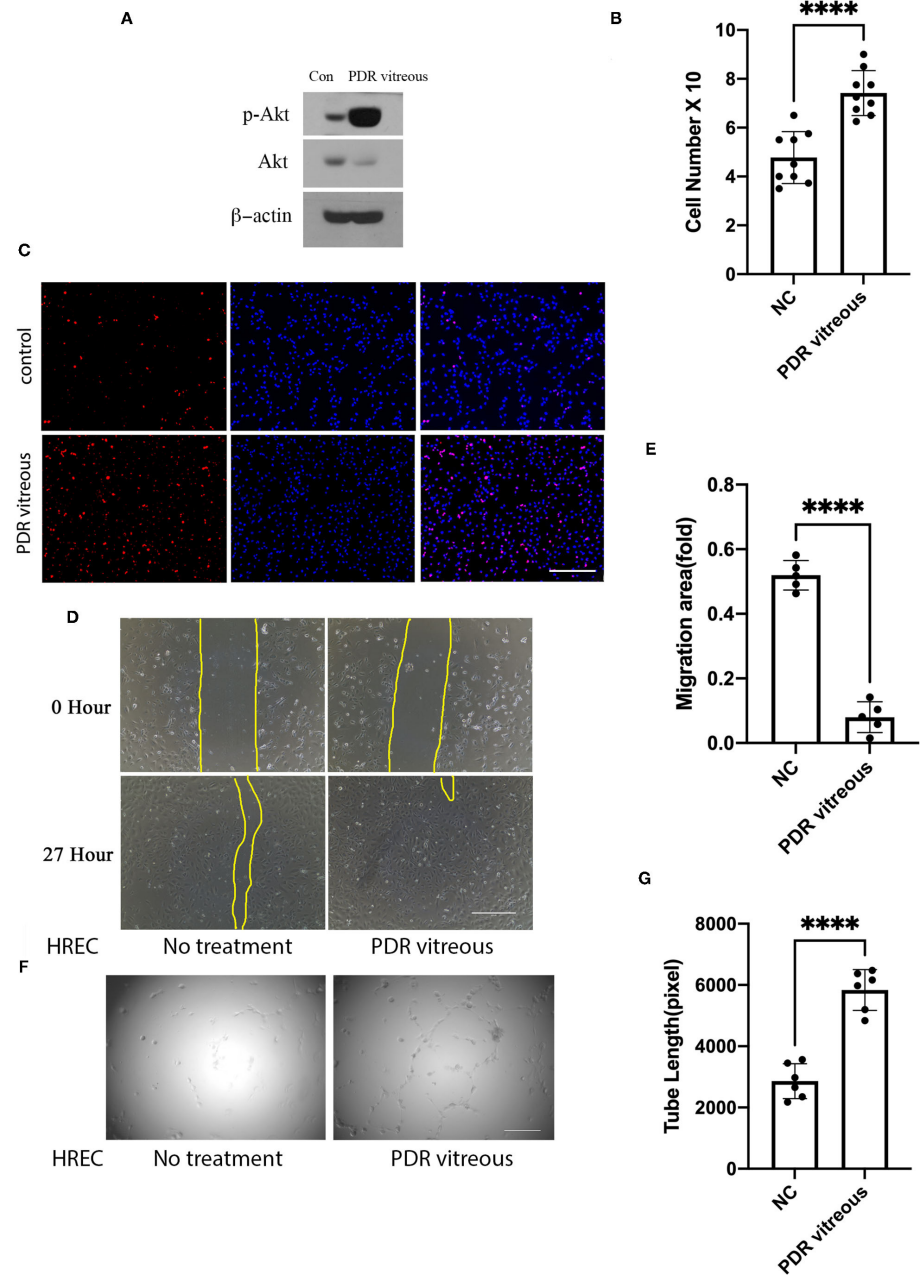
PDR vitreous enhanced proliferation, migration, and tube formation of vascular endothelial cells. **(A)** Western blot analysis of p-Akt in HRECs induced by PDR vitreous. This is representative of three independent experiments. **(B,C)** Proliferation of HRECs induced by PDR vitreous was assessed using two methods. **(B)** Total cell number counting; **(C)** Ki67 staining of proliferative cells. Scale bar: 1,000 μm. **(D,E)** A wound healing assay to measure the migration of HRECs stimulated by PDR vitreous. **(D)** Representative photos, scale bar: 400 μm; **(E)** Bar graphs show five independent experiments. **(F,G)** A matrigel assay to assess the tube formation of HRECs induced by PDR vitreous. **(F)** Representative photos of five independent experiments, Scale bar: 400 μm; **(G)** Bar graphs of tube lengths in five representative photos of three independent experiments. ****means the difference was significant and *p* < 0.0001.

We next assessed if Axl activation could be induced by PDR vitreous in cultured HRECs. As expected, activation of Axl and Akt was heightened in HRECs after stimulation by PDR vitreous whose effect on induction was better than normal vitreous (Figure 2A). These signaling events might transform to angiogenesis-related cellular responses such as proliferation, migration, and tube formation (7–10).

Data from proliferation assays with HRECs showed that there was a significant increase in both the total cell number and Ki-67 (a nuclear protein that is associated with proliferation) positive cell number when these cells were treated with PDR vitreous (Figures 2B,C), indicating that PDR vitreous enhances cell proliferation.

Subsequently, we employed a wound healing assay to assess the migratory ability of HRECs induced by PDR vitreous. The results (Figures 2D,E) showed that PDR vitreous promoted HRECs migration, which is an important feature in angiogenesis.

Subsequently, we evaluated the effect of PDR vitreous on angiogenesis in an *in-vitro* model of a tube formation assay (6). As shown in Figures 2F,G, PDR vitreous heightened the ability of HRECs in forming tubes, suggesting that the signaling pathway of Gas6/Axl might play an important role in these PDR vitreous-enhanced cellular events intrinsic to angiogenesis.

### Suppression of Axl Impedes PDR Vitreous-Induced Cell Proliferation, Migration, and Tube Formation

**Figure 3:**
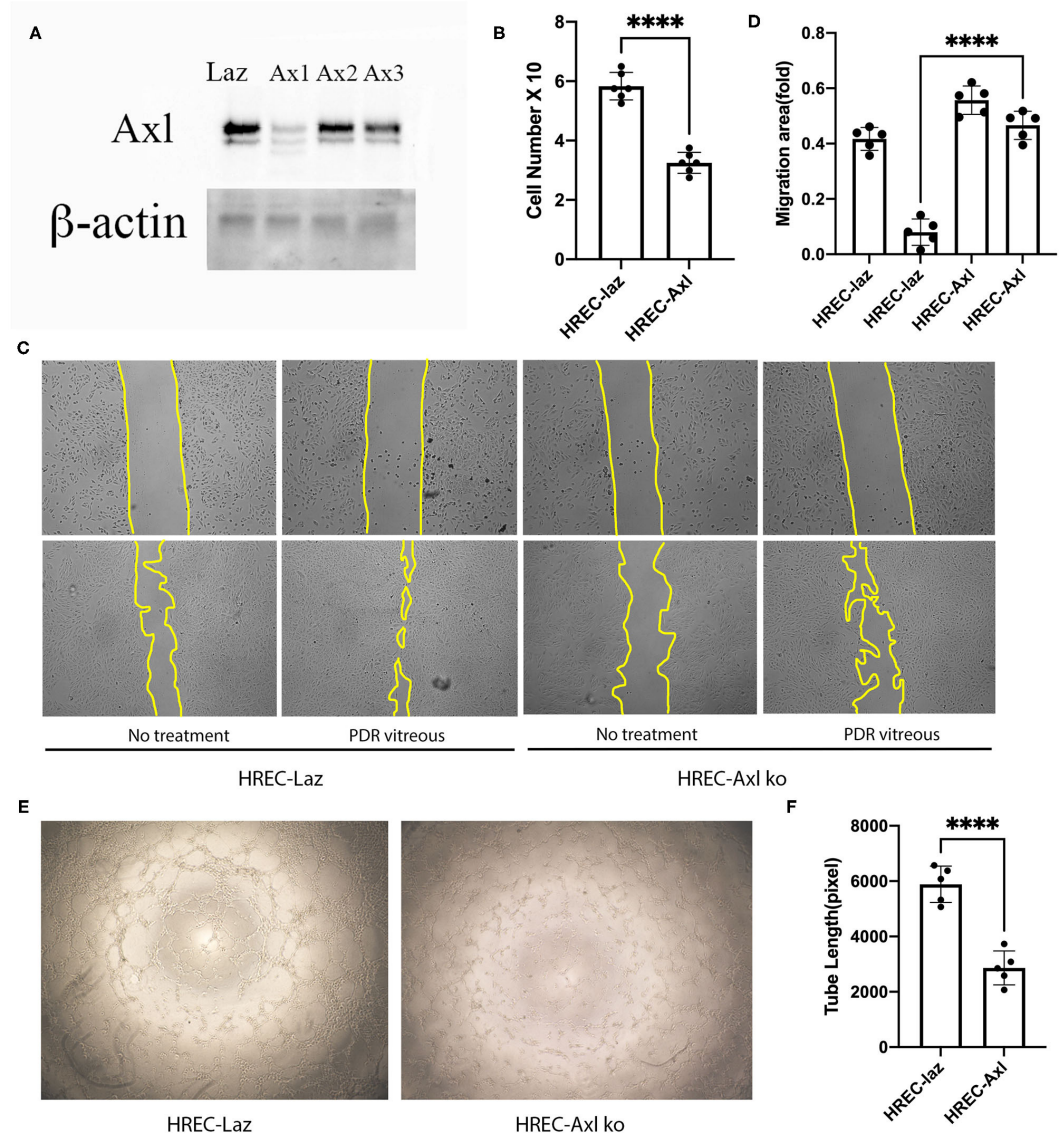
Suppression of Axl suppressed PDR vitreous-induced proliferation, migration, and tube formation. **(A)** Western blot analysis of lysates from HRECs expressing CRISPR/Cas9 targeting *lacZ* or *Axl* using indicated antibodies. This is representative of three independent experiments. **(B)** Proliferation of HRECs expressing CRISPR/Cas9 targeting *lacZ* or *Axl* was induced by PDR vitreous and cell number was counted in a hemocytometer under a light microscope. **(C,D)** Migration of HRECs expressing CRISPR/Cas9 targeting *lacZ* or *Axl* was induced by PDR vitreous in a wound assay. **(D)** Representative photos of the wound areas, scale bar: 400 μm; bar graphs of wound areas in five representative photos from three independent experiments. **(E,F)** A matrigel assay was used to evaluate tube formation of HRECs expressing CRISPR/Cas9 targeting *lacZ* or *Axl* induced by PDR vitreous. **(F)** Representative photos, scale bar: 1,000 μm; Bar graphs of tube lengths in five representative photos of three independent experiments. ****means the difference was significant and *p* < 0.0001.

To investigate the role of Axl in PDR vitreous-induced angiogenesis *in vitro*, expression of Axl was depleted in HRECs by using a CRISPR/Cas9 approach. As shown in Figure 3A, western blot analysis demonstrated that Axl depletion resulted from SpCas9 editing of genomic DNA under the single guide RNA-1 (sgRNA-1) guidance. In addition, we found that depletion of Axl attenuated PDR vitreous-induced cell proliferation (Figure 3B) and decreased the migratory capability of HRECs (Figures 3C,D). Furthermore, Axl removal resulted in a reduction in PDR vitreous-induced tube formation of the HRECs (Figures 3E,F). These results demonstrate that Axl plays a central part in PDR vitreous-stimulated activation of Akt and angiogenesis-related cellular responses of HRECs.

### Inhibition of Axl Prevents PDR Vitreous-Induced Akt Activation and Cellular Responses Related to Angiogenesis

**Figure 4:**
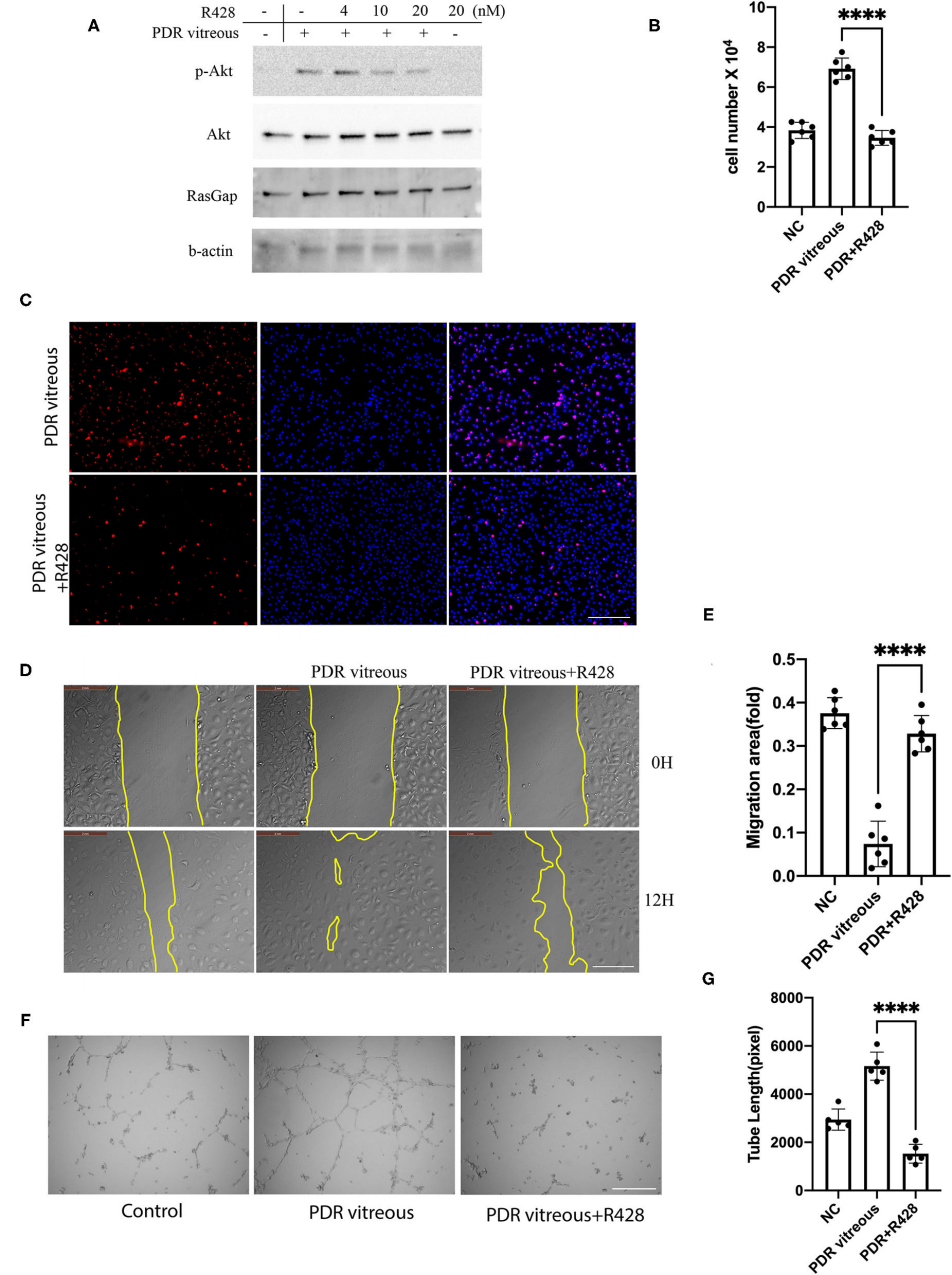
Pharmacological inhibition of Axl blunted PDR vitreous-induced Akt activation, proliferation, migration, and tube formation. **(A)** Western blot analysis of p-Akt in HRECs treated with PDR vitreous and a serial of R428 (an Axl-specific inhibitor) concentrations using indicated antibodies. This is representative of three independent experiments. **(B,C)** Proliferation of HRECs induced with PDR vitreous supplemented with R428 (4 μM) was evaluated by counting cell number **(B)** and Ki67 staining, scale bar: 1,000 μm **(C)**. Bar graphs in **(B)** show the mean ± SD from five independent experiments. **(D,E)** A wound healing assay of migration of HRECs induced by PDR vitreous supplemented with R428 (4 μM). **(D)** Representative of the photographed wound, scale bar: 400 μm; **(E)** Bar graphs show the mean ± SD from five representative figures. **(F,G)** A matrigel assay of HRECs induced by PDR vitreous supplemented with R428 (4 μM). **(F)** Representative photos, scale bar: 400 μm; **(G)** Bar graphs of tube lengths in five representative photos of three independent experiments. ****means the difference was significant and *p* < 0.0001.

We next sought to find if pharmacological inhibition of Axl could block PDR vitreous-induced Akt activation, so that a potential pharmacological inhibitor could be used for treating retinal pathological angiogenesis. R428, a small molecule inhibitor specific for Axl, inhibited Axl at 4 μM without showing obvious toxicity to HRECs. Therefore, we treated HRECs with PDR vitreous along with R428 and found that 4 μM R428 completely blocked PDR vitreous-induced Akt activation (Figure 4A). As expected, R428 at this concentration also inhibited vitreous-stimulated proliferation, migration, and tube formation of HRECs (Figures 4B–G), suggesting that Axl is a potential mediator of retinal angiogenesis.

## Discussion

**Figure 5:**
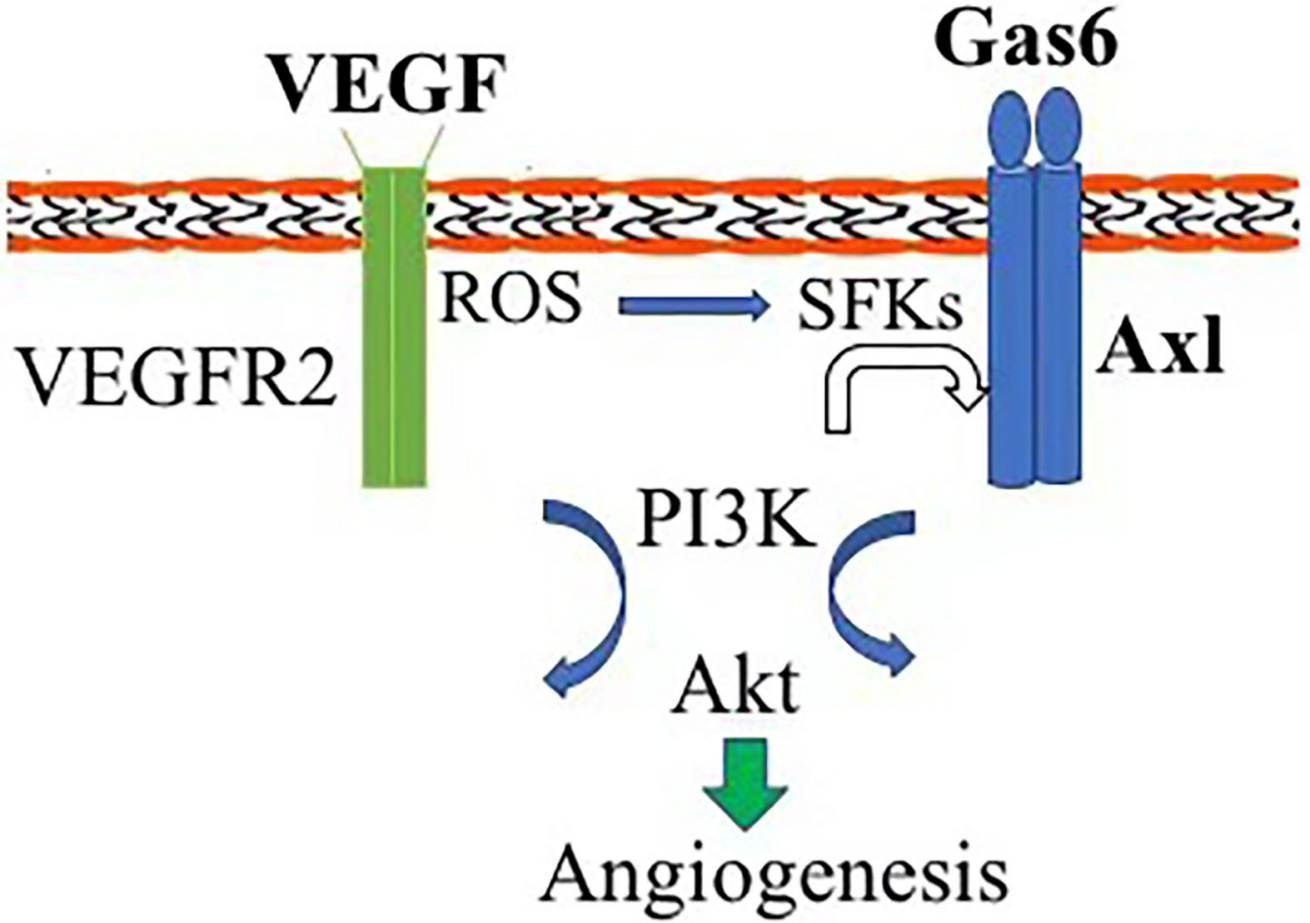
Diagram of Axl contribution to angiogenesis. Axl can be activated by Gas6 direct binding and vascular endothelial growth factor (VEGF) indirectly *via* reactive oxygen species (ROS)/Src-family protein tyrosine kinases, triggering the signaling pathway of PI3K/Akt and initiating pathological angiogenesis.

In this study, we report that Axl, one of receptor tyrosine kinases, is essential for vitreous-induced angiogenesis with a patient *in vitro*. In the vitreous from patients with PDR, levels of VEGF-A are elevated (11). It has been reported that Axl is essential for VEGF-A-dependent activation of the PI3K/Akt signaling, which plays a central role in angiogenesis (3). In addition, Gas6, a traditional ligand of Axl, is also present in such vitreous. Thus, we proposed that there are at least two routes for activating Axl by the PDR vitreous. One is that Axl is directly activated by its ligand Gas6 binding to trigger the signaling pathway of Axl/PI3K/Akt; the other is that Axl is indirectly activated by VEGF-A *via* Vascular endothelial growth factor receptor-2/ROS/SFKs (3) (Figure 5). However, the pathway for Axl plays a predominant role that needs further investigation.

We have previously reported that normal bovine vitreous is also able to activate Axl for engaging activation and angiogenesis of Akt (6). In this study, the vitreous from patients with PDR shows a similar effect to normal bovine vitreous on *in-vitro* angiogenesis; however, PDR vitreous is obviously more relevant to pathophysiological conditions and, thus, these novel findings may provide a significant clue to develop novel approaches to prevent or cure diabetes-related retinal angiogenesis.

We have found that R428, a specific small molecule inhibitor of Axl, inhibits either normal bovine vitreous or PDR vitreous-activated Akt and angiogenesis *in vitro* and *in vivo*. Small molecules have some disadvantages in clinical settings including short half-life, non-specific, etc. Therefore, based on the essential role of Axl in pathological retinal angiogenesis, we may develop novel approaches such as genome-editing Axl for its inactivation in retinal vascular ECs to prevent retinal angiogenesis.

Currently, intravitreal injection of anti-VEGF agents including aflibercept and ranibizumab is mainstream for treating patients with diabetes-related eye diseases; however, there are still numerous such patients who do not respond to the anti-VEGF treatment or develop resistance to the anti-VEGF therapy. In such cases, an anti-Axl therapy may shed a light on those anti-VEGF-resistant patients.

## Methods and Reagents

### Sample and Major Reagents

The Internal Review Committee of Xiangya Hospital approved this study. Research protocols adhered to the Association for Research in Vision and Ophthalmology Statement on Human Subjects and the tenets of the Declaration of Helsinki. All the participants gave a written informed consent prior to surgery and inclusion in this study. Surgical samples were collected at the Department of Xiangya Hospital. Most of the antibodies and reagents in this study were listed in our previous study (6).

### Cell Culture

Human retinal microvascular endothelial cells (Cell Systems, Kirkland, Washington, USA) were grown in an endothelial growth medium (EGM)-2 (Lonza, Walkersville, Maryland, USA) supplemented with a kit of growth factors (Lonza, Walkersville, Maryland, USA). Human embryonic kidney (HEK) 293T cells were cultured in high-glucose (4.5 g/l) Dulbecco's Modified Eagle Medium supplemented with 10% Foetal Bovine Serum. All the cells were cultured at 37°C in a humidified 5% CO_2_ atmosphere (12).

### Deoxyribonucleic Acid Constructs

The transfer vector of lentivirus was constructed by replacing different sgRNA in *BsmBI*-digested lentiCRISPR v2 vector as previously described (6). The three protospacer sequences from Axl loci (NC_000004.12) were 5′-AAGGTTCCTTCACTATCAGG-3′ (A1), 5′-GGGAATATCACAGGTGCCCG-3′ (A2), and 5′-CTTCTACCGGGAAACTGACT-3′ (A3) and the control sgRNA sequence targeting *LacZ* from *Escherichia coli* was (5′-TGCGAATACGCCCACGCGATGGG-3′) (13). All the clones were confirmed by DNA sequencing using a primer 5′-GGACTATCATATGCTTACCG-3′ from the sequence of U6 promoter that drives expression of sgRNAs. Both the synthesis of primers and oligos and sequencing of PCR products and clones were performed at Sangon Biotech (Shanghai, China). All the plasmids used were purchased from Addgene (Cambridge, Massachusetts, USA).

### Production of Lentivirus

The procedure for lentivirus production was described in detail in our previous publication (14). Lentiviruses were produced by triple transfection of HEK 293T cells with pLentiCRISPRv2, the lentiviral packaging plasmid which encod HIV-1 Gag, Pol, Tat and Rev proteins, and pVSV-G [catolog number 52,961, 12,260, and 8,454 from Addgene (Cambridge, Massachusetts, USA), respectively] using lipofectamine 3000. After harvest, viruses were concentrated by centrifuging in a JA17 rotor (Beckman Coulter, Brea, California) at 25,000 g for 90 min at 4°C. The concentrated virus was resuspended in 300 μl of sterile TNE (50 mM Tris, Ph 7.8, 130 mM sodium chloride, and 1 mM Ethylenediaminetetraacetic acid) with gentle rotation overnight at 4°C. Next, these dissolved retroviruses were tittered for infecting HRECs in combination with 8 μg/ml polybrene (Sigma-Aldrich Corporation, St Louis, Mosby, USA) or kept at −80°C (12, 15, 16). The infected cells were selected in media with puromycin (Sigma-Aldrich Corporation, St Louis, Mosby, USA) (0.5 μg/ml) and the resultant cells were examined by Western blot analysis (12, 15, 16).

### Western Blot

Human retinal microvascular endothelial cells were seeded in a 24-well plate at 70% confluence and then starved for 6–8 h in media deprived from serum and growth factors. Subsequently, the starved cells were pretreated with PDR vitreous (200 μl/ml) for 30 min and the control group was pretreated with boiled PDR vitreous. In certain experiments, cells were treated with different inhibitors for 30 min. All the cells were lysed in Radioimmunoprecipitation assay in the presence of a protease inhibitor (MedChemExpress shanghai, China). The protein-transferred membranes were blocked by 5% fat-free milk dissolved in Tris Buffered Saline/0.05% Tween-20 for 30 min and incubated with first antibodies (1:1,000) overnight at 4°C. After thoroughly washing, the membranes were incubated with horse radish peroxidase-conjugated secondary antibodies (1:5,000) and visualized using LumiBest enhanced chemiluminescence. β-actin and total Akt were served for normalization across the samples. Experiments were repeated at least three times and blot signal intensity was determined by densitometry using the National Institutes of Health ImageJ software (12).

### Cell Proliferation Assay

Human retinal microvascular endothelial cells were seeded at a density of 2 × 10^4^ cells/well in 24-well plates and counted in a cell number counter after 48 h of continuous treatment with endothelial cell growth media-2 or PDR vitreous (1:3 dilution in EGM-2). At least three independent experiments were performed as described previously (6). In addition, immunofluorescent staining for Ki67 (rabbit anti-Ki67; Proteintech, Rosemont, IL US) for marking proliferating cells was performed as previously described (17).

### Scratch-Wound Migration Assay

Migration was assessed with a scratch-wound assay (18). Once cells reached 80% confluence in 24-well plates, they were starved for 4 h. After the cell monolayer was scraped with a sterile pipette tip (200 μl), the cells were washed twice to remove detached cells. One scratch was generated per well and imaged on an Leica imaging system every 6 h for 48 h. Images were analyzed by measuring the number of pixels in the wound area using Adobe Photoshop (Adobe Systems, San Jose, California, USA) and analyzed using the ImageJ software (3).

### Tube Formation Assay

This assay was performed as previously described (19). A total of 15,000 HRECs were placed onto wells precoated with basement membrane extract (R&D Systems, Minneapolis, MN, US), which was from the storage at −80°C and thawed overnight at ice. Cells were imaged 4–6 h after cell plating and tube formation was quantified for the total length using the Angiogenesis Analyzer plugin for the ImageJ software (National Institutes of Health) (20).

### Statistical Analysis

Results are presented as mean ± SD. The Student's *t*-test was performed for comparisons between two groups and the one-way ANOVA (Kruskal–Wallis test) was used for comparisons among the multiple groups. *p* < 0.05 was considered as statistically significant.

## Data Availability Statement

The original contributions presented in the study are included in the article/Supplementary Material, further inquiries can be directed to the corresponding author/s.

## Ethics Statement

The studies involving human participants were reviewed and approved by Xiangya Hospital, Central South University. The patients/participants provided their written informed consent to participate in this study.

## Author Contributions

WW performed the study and wrote the manuscript. HX provided patient-derived vitreous and PDR membrane. ZM and JZ contributed to the quantification of the experiment data. SX, XX, and HL analyzed the data and revised the manuscript. All authors contributed to the article and approved the submitted version.

## Funding

This study was supported by the grant National Natural Science Foundation of China (grant nos. 81900893 to WW, 81974137 to SX, 81974134 to XX, 82070989 to HL, and 82002373 to JZ), the Science and Technology Plan Project of Hunan Province (grant no. 2019RS2011) to WW, the Youth Science Foundation of Xiangya Hospital (grant no. 2019Q19 to JZ), and the Natural Science Foundation of Hunan Province (grant nos. 2021JJ41030 to WW, 2020JJ5958 to JZ, and 2019JJ40507 to SX).

## Conflict of Interest

The authors declare that the research was conducted in the absence of any commercial or financial relationships that could be construed as a potential conflict of interest.

## Publisher's Note

All claims expressed in this article are solely those of the authors and do not necessarily represent those of their affiliated organizations, or those of the publisher, the editors and the reviewers. Any product that may be evaluated in this article, or claim that may be made by its manufacturer, is not guaranteed or endorsed by the publisher.
