# Erratum: Axl Is Essential for *in-vitro* Angiogenesis Induced by Vitreous From Patients With Proliferative Diabetic Retinopathy

**DOI:** 10.3389/fmed.2022.857417

**Published:** 2022-02-07

**Authors:** Frontiers Production Office

**Affiliations:** Frontiers Media SA, Lausanne, Switzerland

**Keywords:** PDR vitreous, GAS6, Axl, CRISPR/Cas9, R428, HRECs

Due to a production error, there was an error in affiliations 1 and 4. Instead of “The Second Xiangya Hospital of Central South University,” it should be “Xiangya Hospital of Central South University.” The publisher apologizes for this mistake. The original version of this article has been updated.

