# Supplementary figures and images for: Axl Is Essential for in-vitro Angiogenesis Induced by Vitreous From Patients With Proliferative Diabetic Retinopathy
Source: Front Med (Lausanne). 2021 Dec 23;8:787150. doi: 10.3389/fmed.2021.787150 (PMC8734562; doi:10.3389/fmed.2021.787150)

## Slide 1
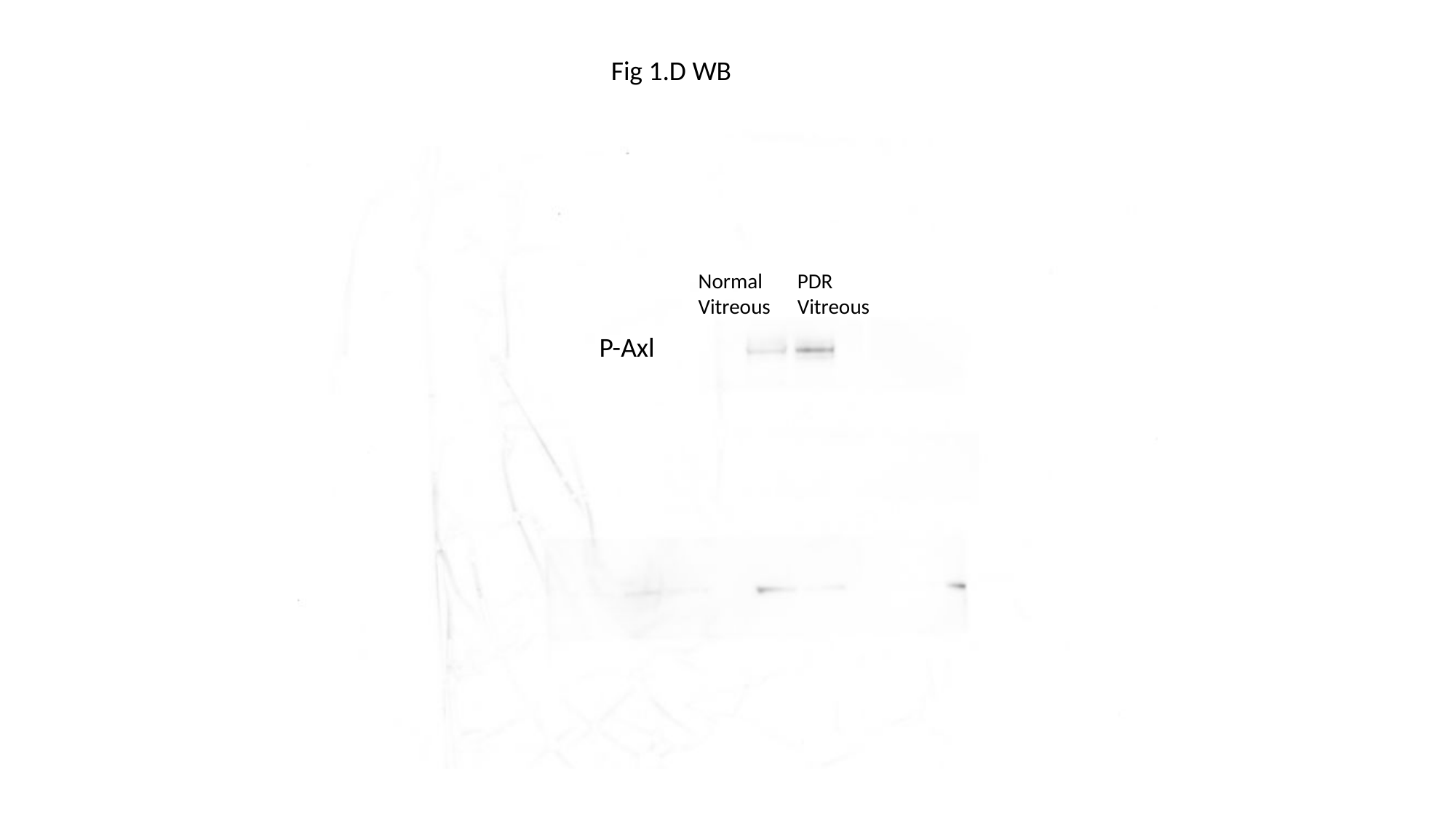

Fig 1.D WB
Normal
Vitreous
PDR
Vitreous
P-Axl

## Slide 2
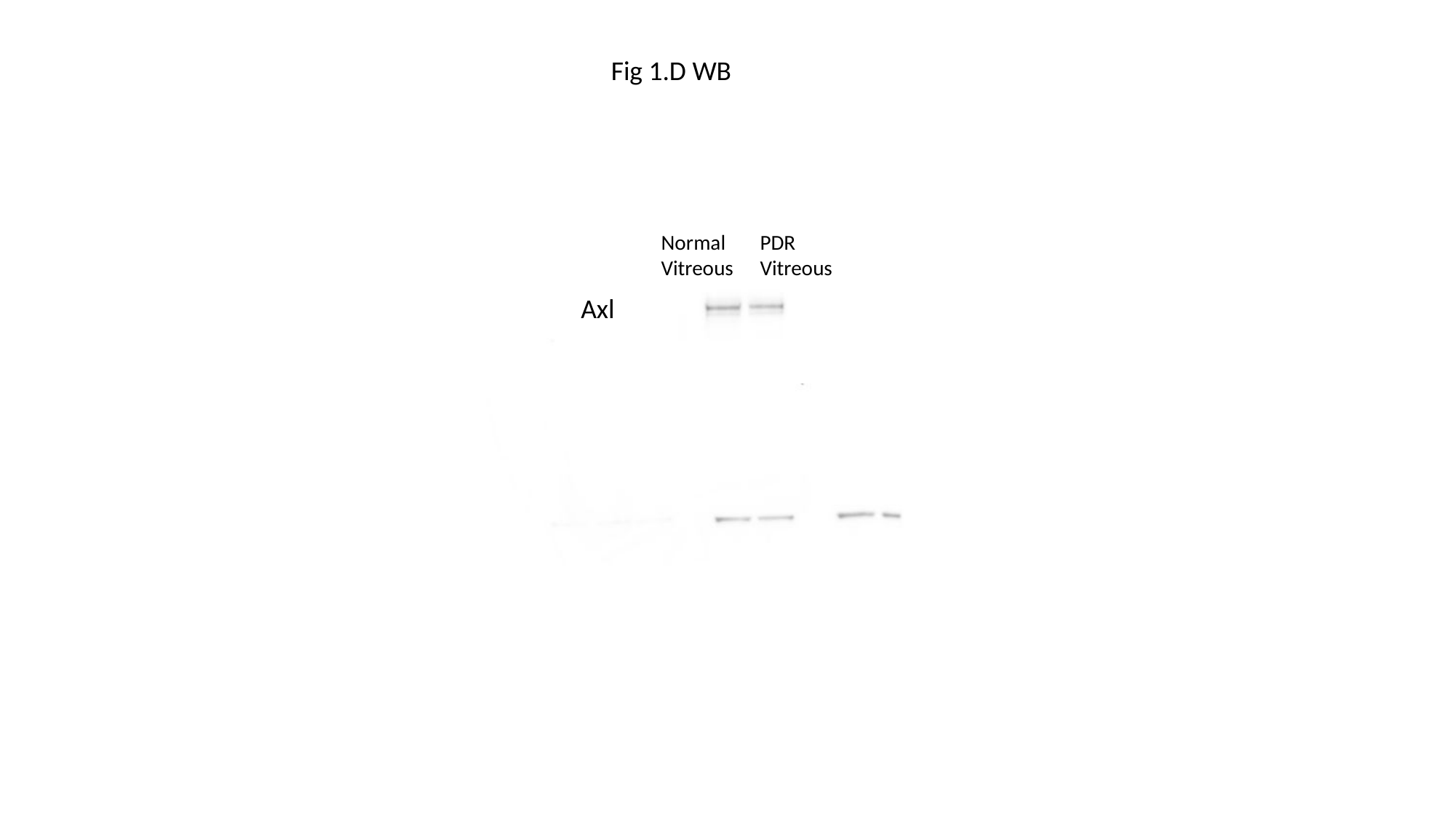

Fig 1.D WB
Normal
Vitreous
PDR
Vitreous
Axl

Supplement: Supplementary file 4 [file Presentation_1.PPTX]

## Slide 1
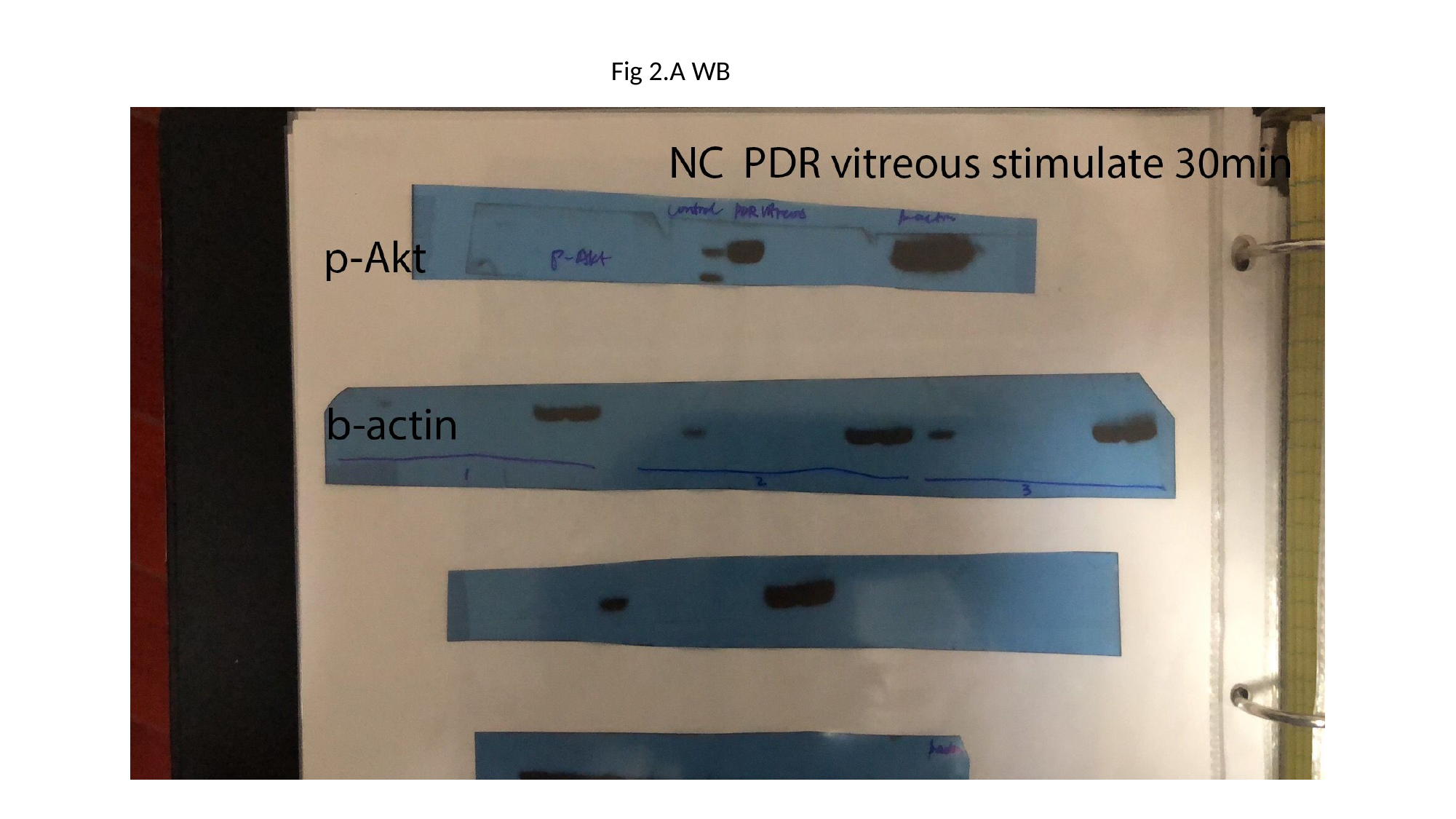

Fig 2.A WB

Supplement: Supplementary file 5 [file Presentation_2.PPTX]

## Slide 1
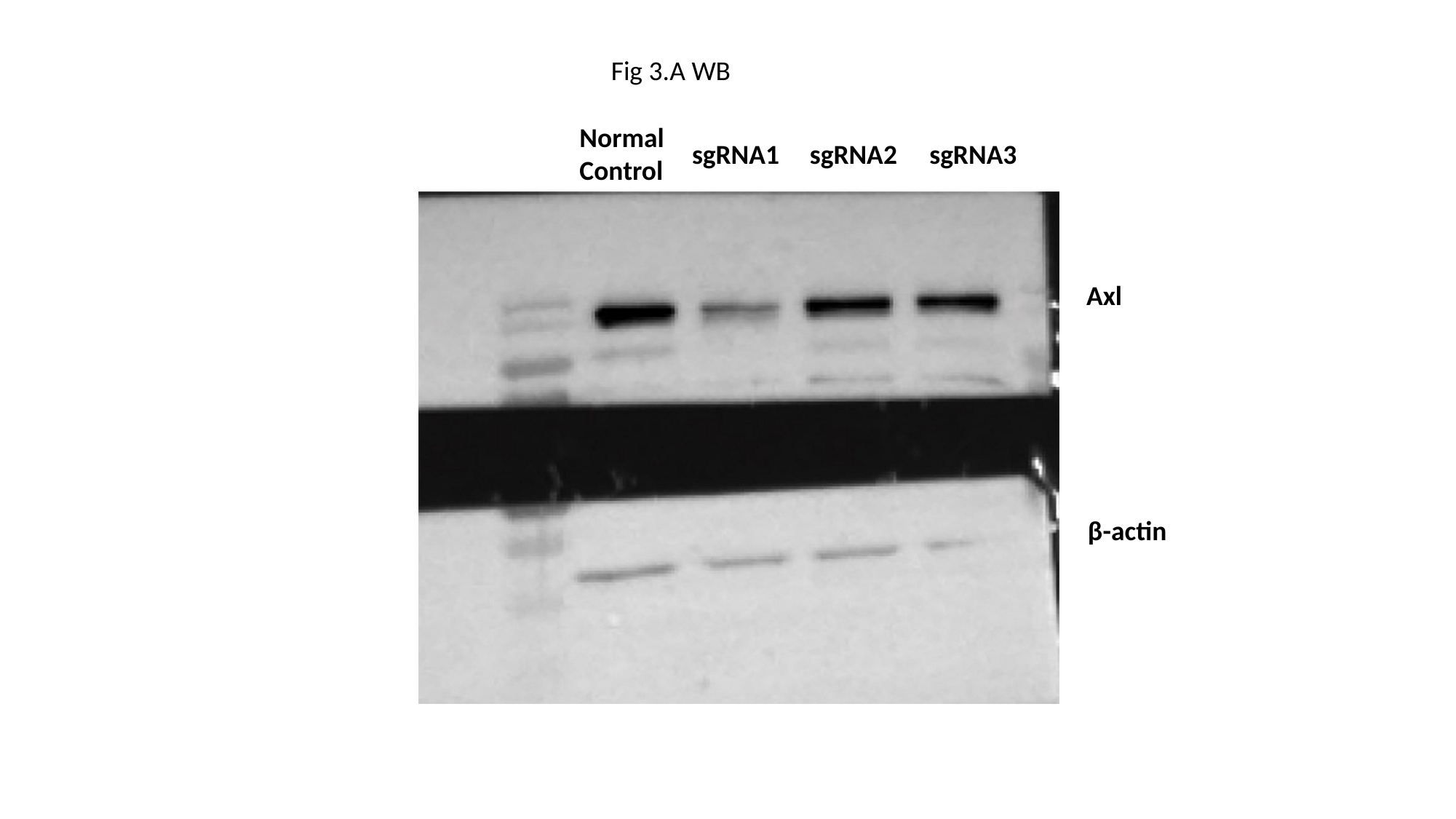

Fig 3.A WB
Normal
Control
sgRNA3
sgRNA1
sgRNA2
Axl
β-actin

Supplement: Supplementary file 6 [file Presentation_3.PPTX]

## Slide 1
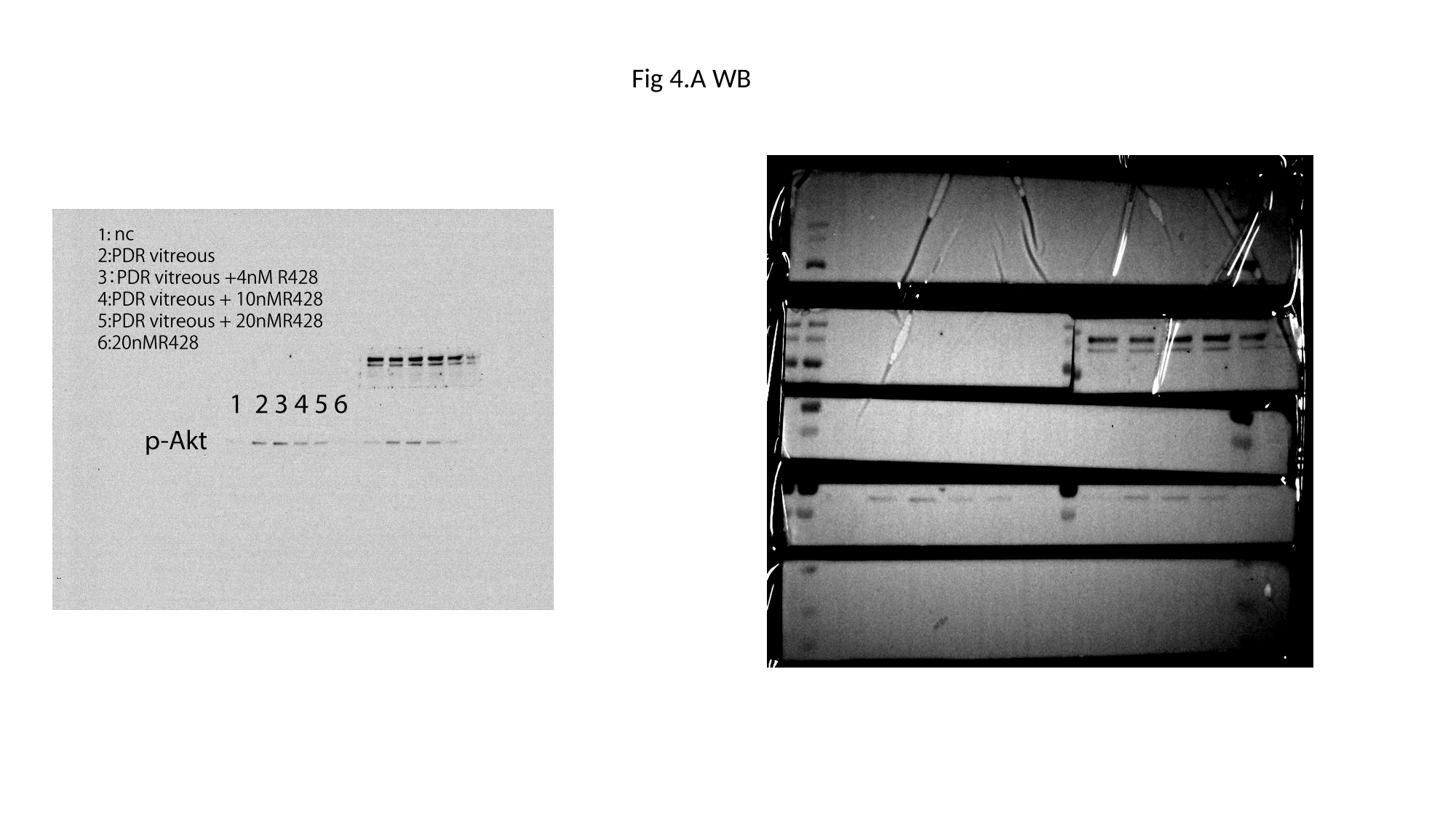

Fig 4.A WB

Supplement: Supplementary file 7 [file Presentation_4.PPTX]

## Slide 1
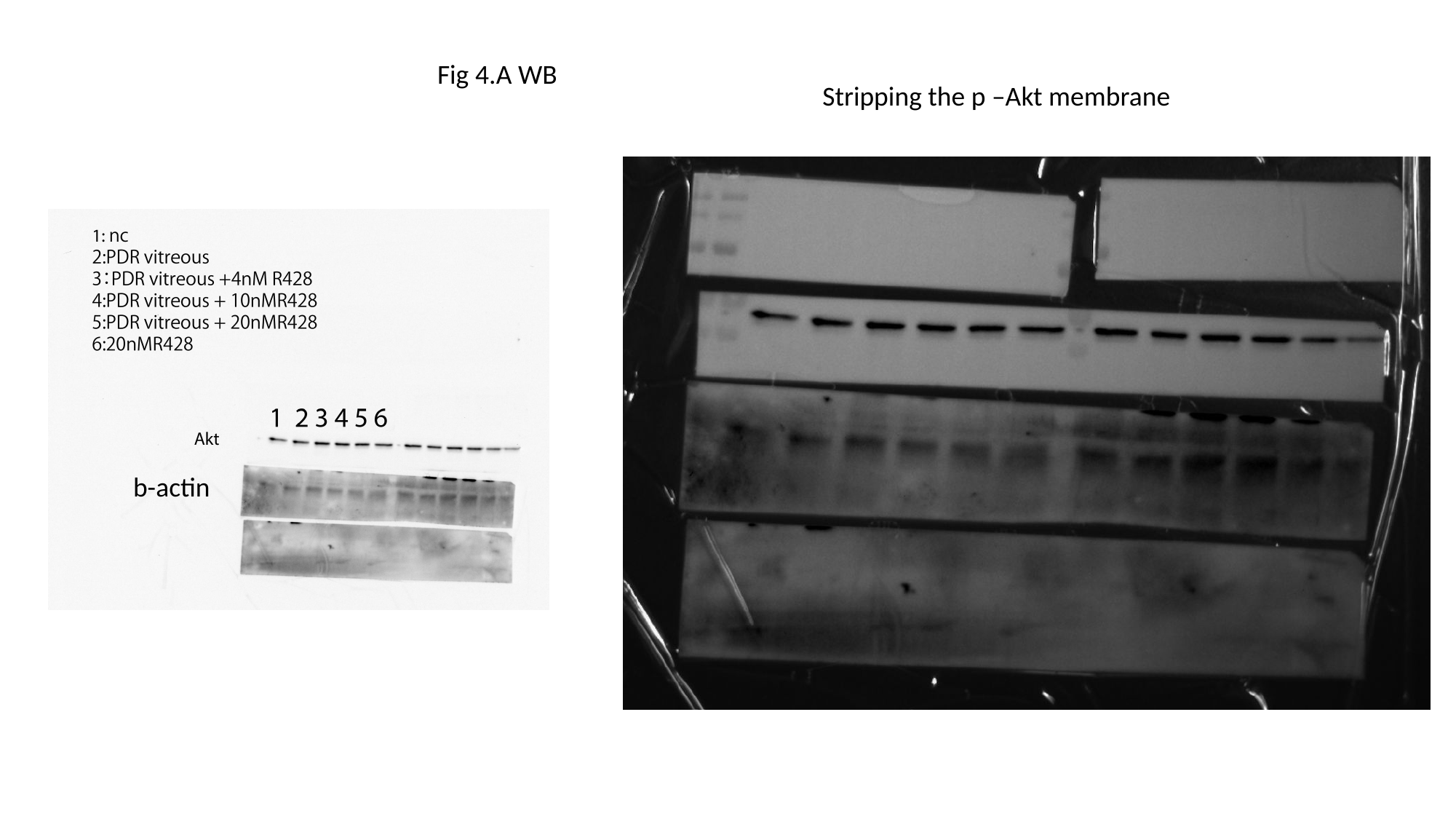

Fig 4.A WB
Stripping the p –Akt membrane
b-actin

Supplement: Supplementary file 8 [file Presentation_5.PPTX]

## Slide 1
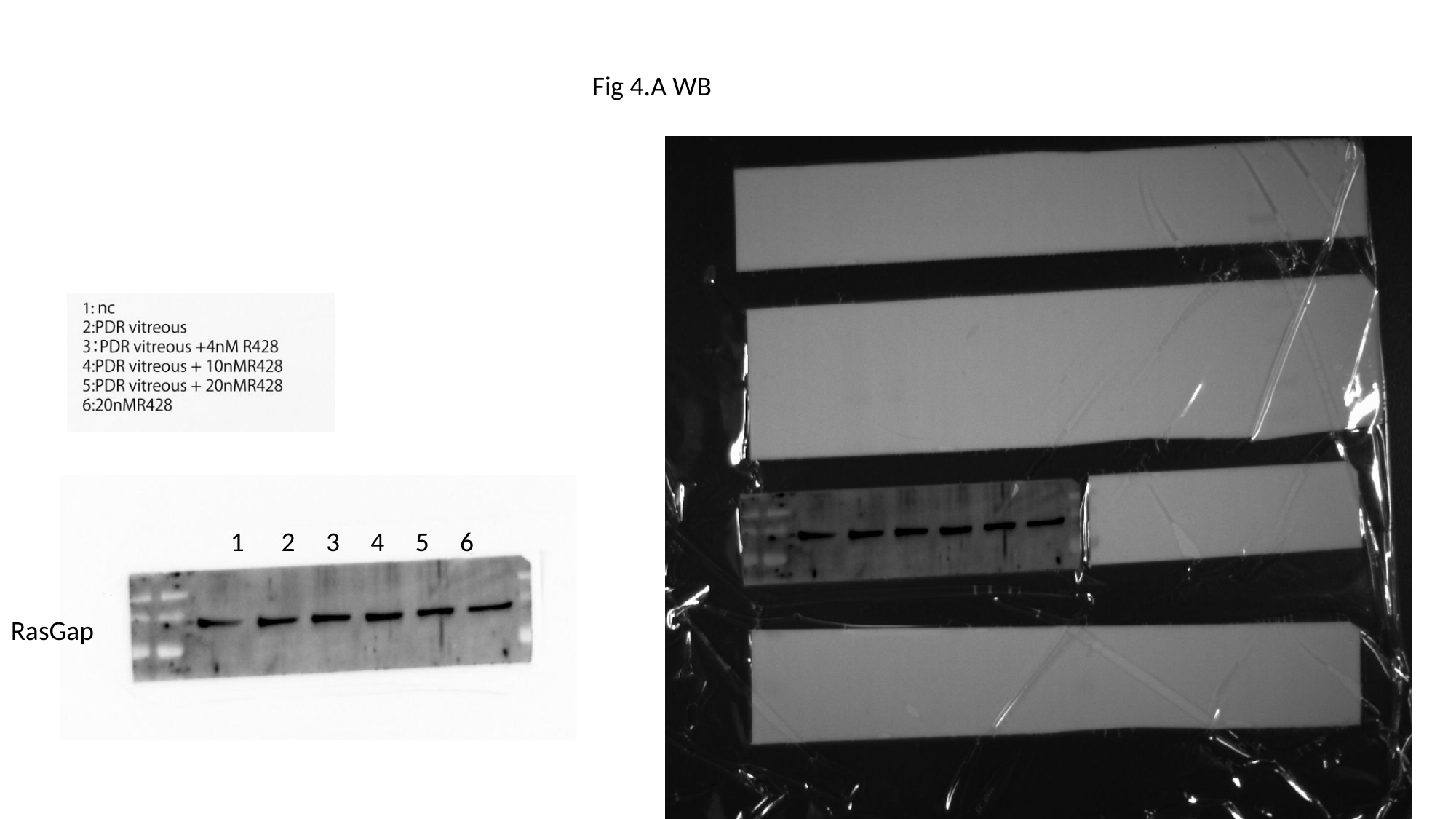

Fig 4.A WB
1 2 3 4 5 6
RasGap

Supplement: Supplementary file 9 [file Presentation_6.PPTX]

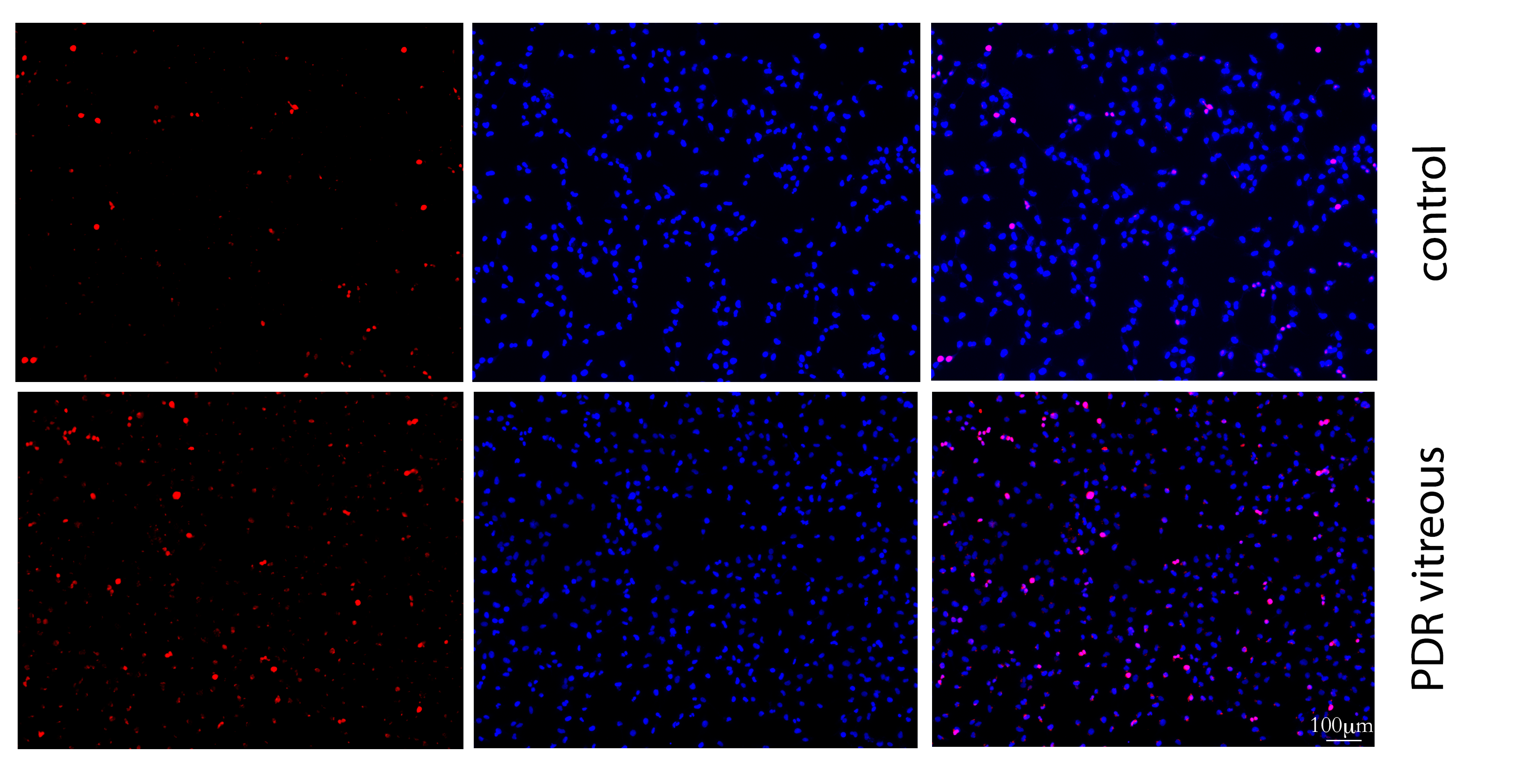

Supplement: Supplementary file 10 [file Image_1.TIF]

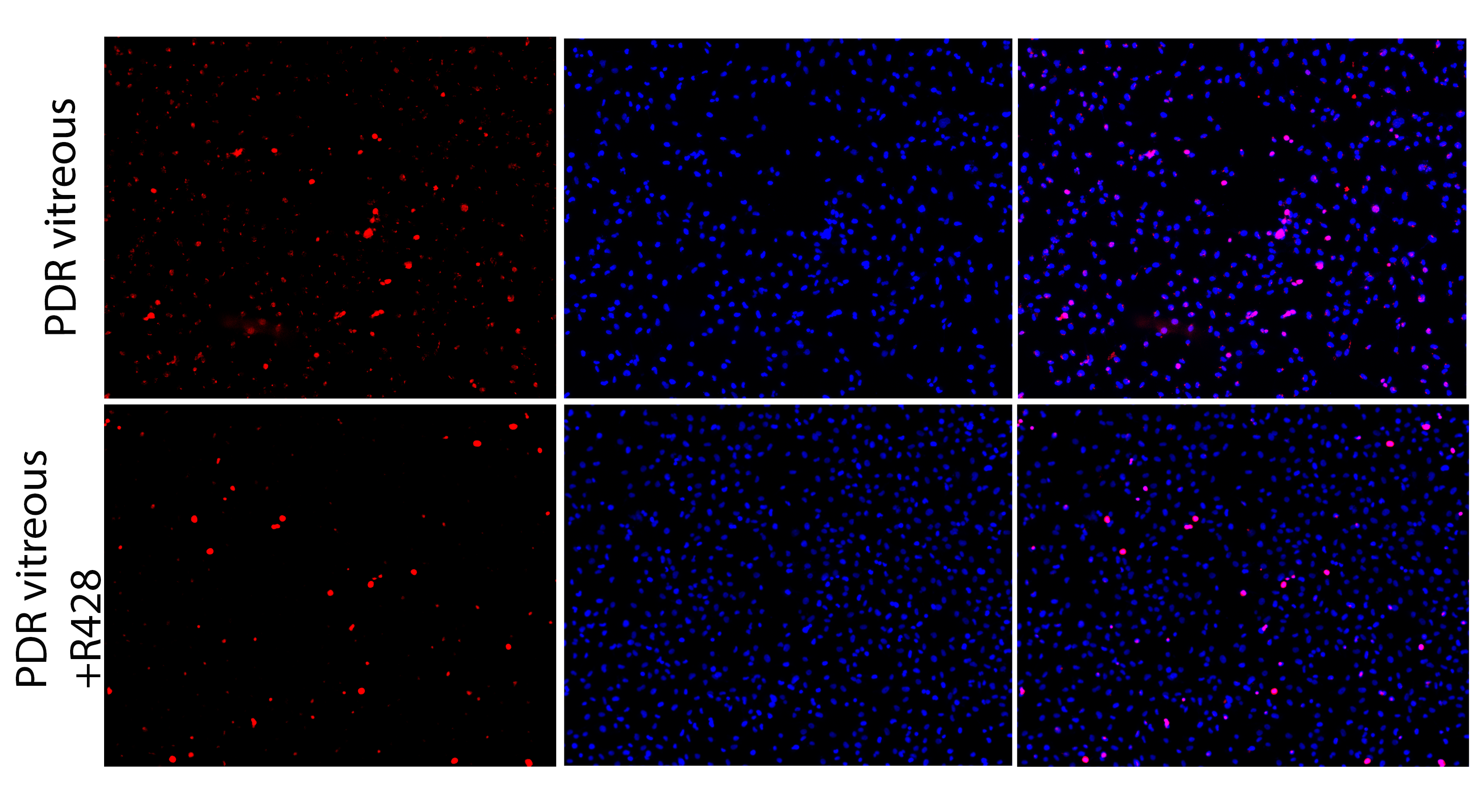

Supplement: Supplementary file 11 [file Image_2.TIF]

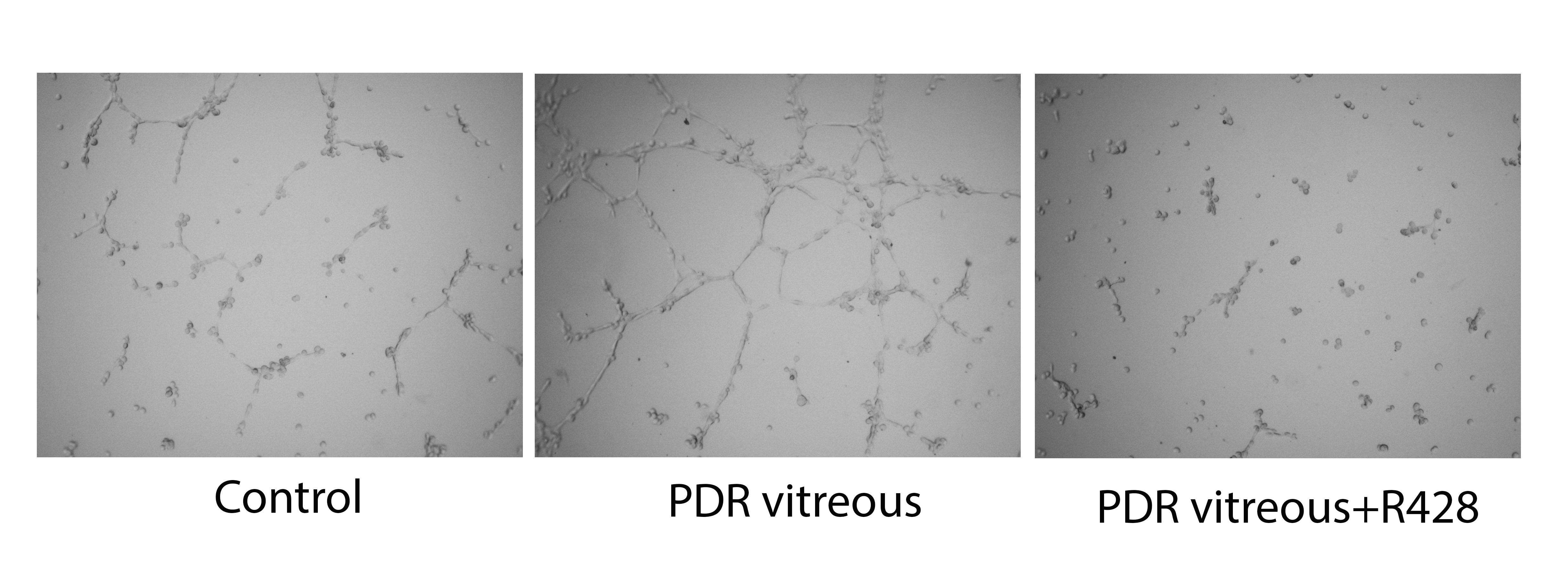

Supplement: Supplementary file 12 [file Image_3.TIF]

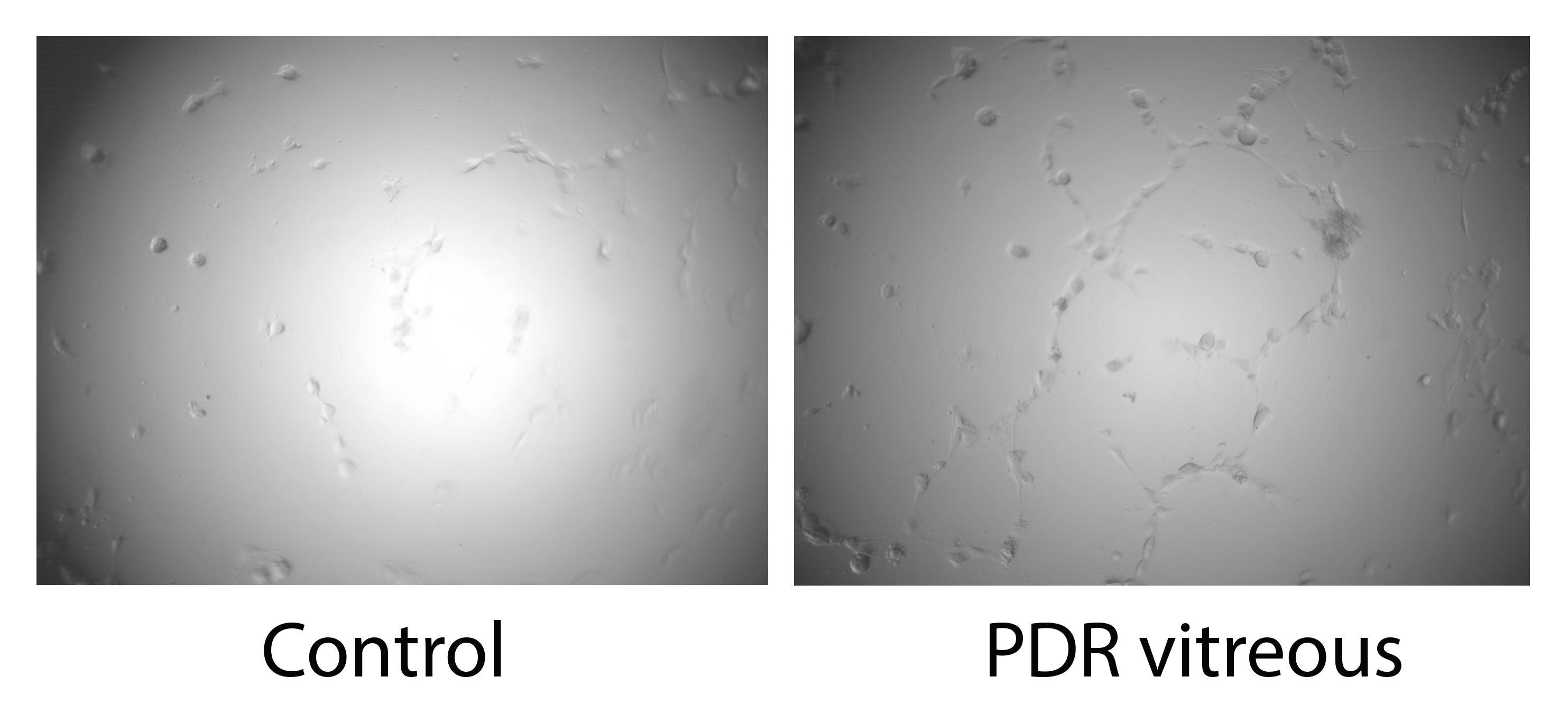

Supplement: Supplementary file 13 [file Image_4.TIF]

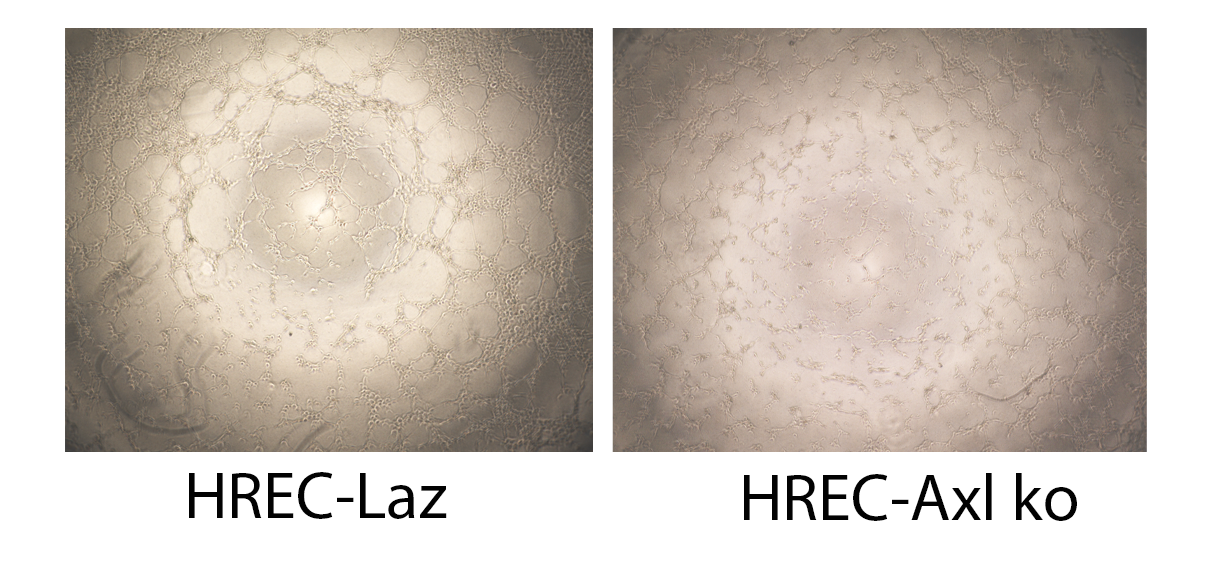

Supplement: Supplementary file 17 [file Image_8.TIF]

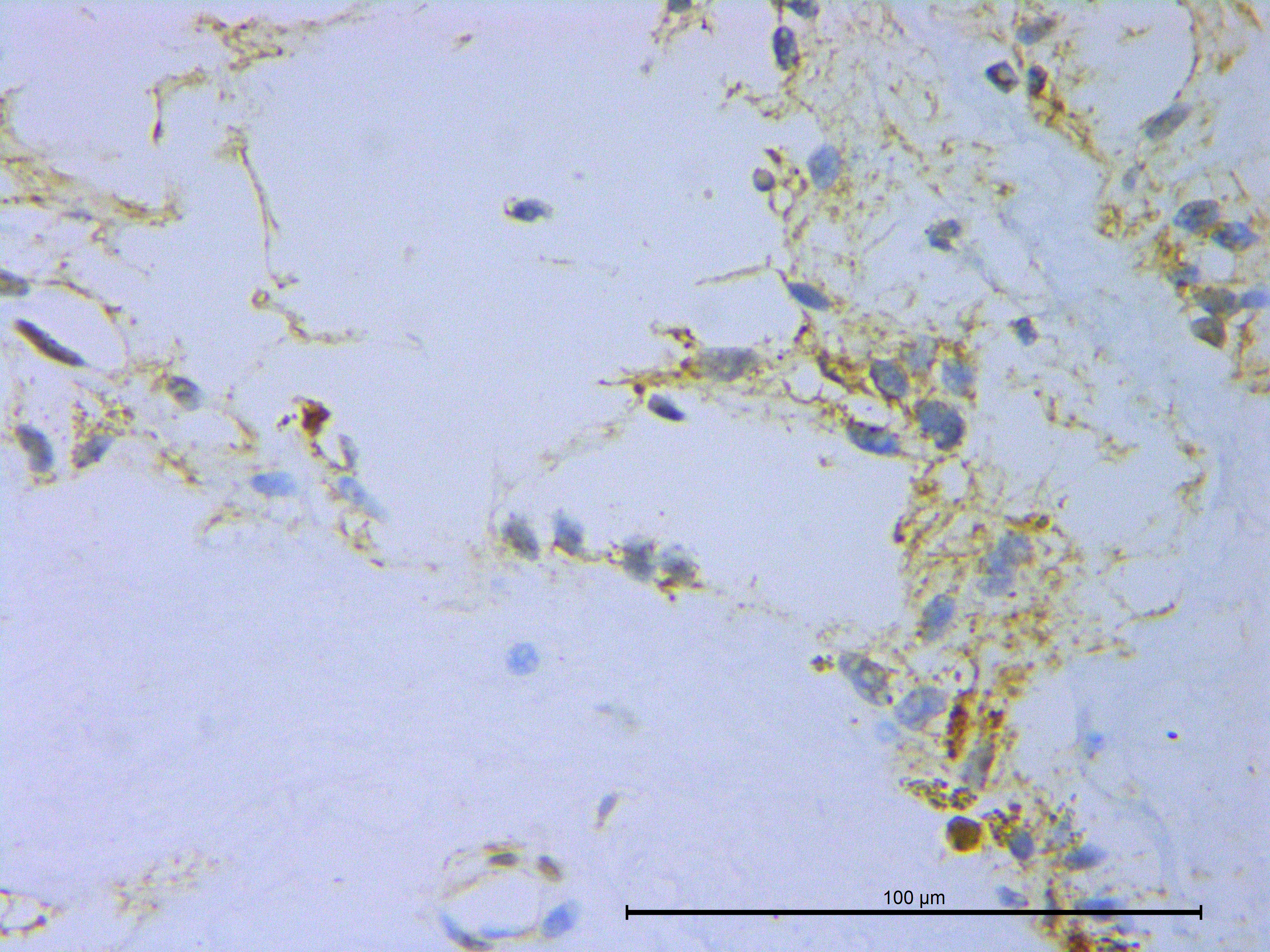

Supplement: Supplementary file 18 [file Image_9.JPEG]

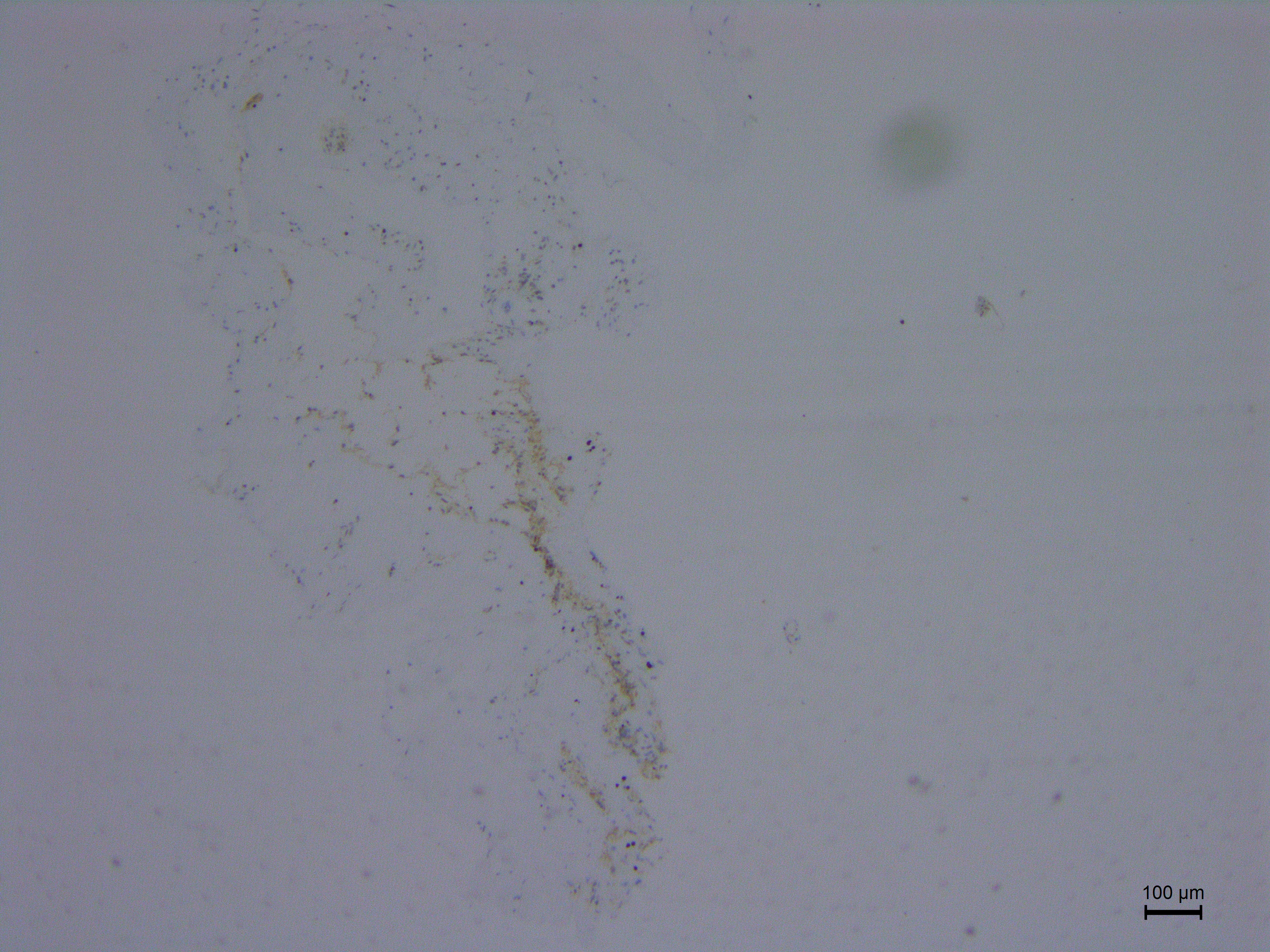

Supplement: Supplementary file 19 [file Image_10.JPEG]

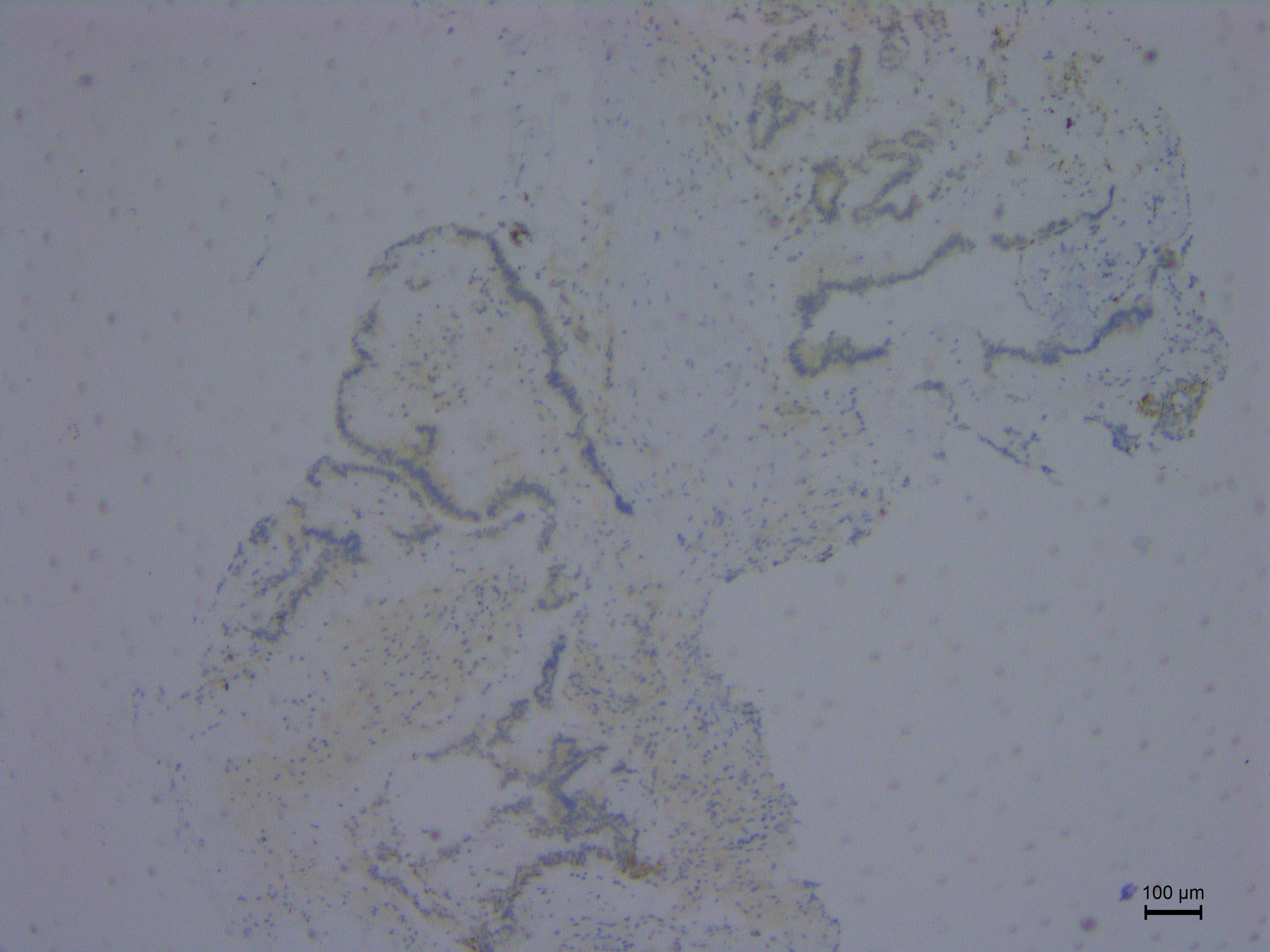

Supplement: Supplementary file 20 [file Image_11.JPEG]
